# Book Reviews

**Published:** 1888-03

**Authors:** 


### BOOK-REVIEWS.

GYNÆCOLOGICAL DIAGNOSIS, GENERAL GYNÆCOLOGICAL THERAPEUTICS, by R. CHROBAK, M. D.; and ELECTRICITY IN GYNÆCOLOGY AND OBSTETRICS, by EGBERT H. GRANDIN, M. D.—Comprising Vol. V, of the Cyclopædia of Obstetrics and Gynæcology. New York: William Wood & Company. 1887. Chicago: W. T. Keener.

The editor frequently refers to "the general practitioner" in a manner which leads one to infer that this volume is intended especially for his guidance. It will be most useful to that class of physicians, but some others may also find its pages more than simply interesting reading. The Vienna author has succeeded very well in limiting his discussion to the subjects implied by the title, and the style of treatment is characteristically encyclopædic.

The second part of the volume will probably be the most useful, since what it contains is not unknown to those doctors who are diligent readers of current medical literature. The subject-matter will be new to many of the subscribers to the Library. Physicians who are unfamiliar with the use of electricity in gynæcological and obstetrical practice will be surprised by the statements in this volume, and highly gratified if they will make intelligent attempts to verify them. The author clearly recognizes and frankly admits that electricity is still used largely empirically in this field, and he is to be congratulated in having made a most excellent résumé of the present knowledge of its usefulness and of the methods of its application. E. W.

DISEASES OF THE OVARIES. By DR. R. OHLSHAUSEN. Edited by EGBERT H. GRANDIN, M. D. Volume VIII, of the Cyclopædia of Obstetrics and Gynæcology. Pp. v and 414. New York: William Wood & Company. Chicago: W. T. Keener. 1887.

Any expectations in regard to the probable thorough and satisfactory character of this volume in the *Cyclopædia*, based upon the reputation of its distinguished author, must be considered as having been fully met in this book. It is, of course, impossible for a treatise on a subject in which so much active and accurate work is being done as in the field of surgery of which this book treats, to do more than report the history, pathology, diagnosis, and treatment up to the date of its publication, and he will be a carping critic who fails to find in this book a careful, accurate, and intelligent résumé of the present knowledge of the subject.

Since upon its successful performance so much depends, general interest will centre upon what is said of the operation for extirpation of the ovaries. The credit of the first operation is given to Ephraim McDowell, of Kentucky, performed in 1809. Nathan Smith, of Connecticut, was the next operator, and his first case, in ignorance of McDowell's work, was performed in 1821. The first English operation was by Lizars, in 1824; the first German by Chrysmar, about 1820; the first French by Woyerkowsky, in 1844. The author thinks that the most decisive advance in the history of ovariotomy was made by Spencer Wells, and he links with his name those of Washington Atlee, Keith, Baker Brown, and Kœberlé. As specimens of the author's opinions upon important, and as yet perhaps unsettled points, the following quotations may be of interest here:

"With strict antisepsis, at all events, the length of the incision exerts no notable influence upon the result of the operation." "I must earnestly protest against flooding the peritoneum with large amounts of fluid. * * * * I have so often observed symptoms of shock immediately after irrigation with a watery solution or thymol (1–10 per cent.) that the connection between the irrigation and the shock is undoubted. Some of these patients did not recover from the collapse, and I am decidedly of the opinion that I have repeatedly lost patients as the result of these irrigations." "There are only two difficulties which occasionally prove insuperable:

"1. The extension of a malignant neoplasm of the ovary to adjacent tissues and organs.

"2. Sub-serous development of the tumor in its most aggravated form.

"On the other hand I recommend, when a larger or smaller portion of the tumor must be left behind, that it be simply replaced in the abdominal cavity and the latter completely closed. Even if the tumor, many of whose cysts have been opened, now communicates freely with the abdominal cavity, and the whole condition presents a filthy appearance, nevertheless there is no danger of sepsis or peritonitis if the operation is performed antisceptially." E. W.

STERILITY; DEVELOPMENTAL ANOMALIES OF THE UTERUS, by P. MÜLLER, M. D.; and THE MENOPAUSE, by E. BÖRNER, M. D. Edited by EGBERT H. GRANDIN, M. D. Vol. XI, of the Cyclopædia of Obstetrics and Gynæcology. New York: William Wood & Company. 1887. Chicago: W. T. Keenen.

The impression which this title produces is that the volume has the least practical value of any in the series. Whether this proves true for individual readers will depend largely upon the nature of their work. There is, however, an agreeable surprise in store for those who will take the trouble to read the book. The first part, that on Sterility, is treated with characteristic German thoroughness, and contains about everything either of value or of interest known to the profession.

The author of the part treating of the Menopause states that he has endeavored to make the treatise of practical value to physicians, and claims that he has had exceptional facilities for the study of his subject.

HANDBOOK OF PRACTICAL MEDICINE. By DR. HERMANN EICHHORST, Professor of Special Pathology and Therapeutics and Director of the University Medical Clinic in Zurich. Volume IV. Diseases of the Blood and Nutrition and Infectious Diseases. Seventy-four wood engravings. Volume IV of Wood's Medical Library for 1886. New York: William Wood & Company. Chicago: W. T. Keener.

The translator's work of the final volume of Professor Eichhorst's valuable work on practical medicine has been as well done as that of the preceding volumes. Professor Eichhorst's work is too well known to need praise here. The fortunate possessor of this Practice of Medicine will thoroughly appreciate it, and thank the publishers for placing it within the reach of American physicians. F. B.

DISEASES OF THE TUBES, LIGAMENTS, PELVIC PERITONEUM AND PELVIC CELLULAR TISSUE; Extra-Uterine Pregnancy, by L. BANDL, M. D.; and DISEASES OF THE EXTERNAL FEMALE GENITALS; LACERATIONS OF THE PERINEUM, by P. ZWEIFEL, M. D. Edited by E. H. GRANDIN, M. D. Volume XII, of the Cyclopædia of Obstetrics and Gynæcology. New York: William Wood & Company. 1887. Chicago: W. T. Keener.

This volume, which closes the series, calls for no special comment beyond the statement that it is no less admirable than the other numbers of the Cyclopædia, and the few remarks appropriate in this connection may relate to the publication as a whole. The first four volumes, those which comprise the translation of Charpentier's Obstetrics, are perhaps the most important of the series, as this is beyond question the most complete treatise upon the subject. The fact is so striking and unquestionable that attention is again called to the greatly enhanced value which the editorial insertions give this part of the series. This occurs for two reasons: 1, the original is unfair in its treatment of German views; and, 2, upon many disputed points in the practice of obstetrics the opinions which may be here classed as the *not-French*, are both entitled to a fairer consideration than the author gives them, and are practically superior. The editorial treatment of these two points leaves little to be desired.

The merit of the several volumes of the Cyclopædia is very uniform, and the interest which they possess for the profession at large will vary much more with the particular subject discussed and with the eminence of the author than by reason of the special ability and thoroughness of the discussion. Gynæcology is undergoing such rapid development that a treatise of this kind must soon cease to represent the best opinions upon many topics, but the work is truly cyclopædic and a credit to the publishers. The great merit of the work, together with the very low price at which it is sold, will certainly insure a very large sale.

The only serious fault, the defective illustrations of the earlier numbers, can be easily corrected in the new editions, many of which ought to be called for. E. W.

A PRACTICAL TREATISE ON RENAL DISEASES AND URINARY ANALYSIS. By WILLIAM HENRY PORTER, M. D., Professor of Clinical Medicine and Pathology in the New York Post-Graduate Medical School and Hospital; Curator to the Presbyterian Hospital. One Vol.; 360 pages, 100 illustrations. New York: William Wood & Co. Chicago: W. T. Keener.

Professor Porter presents, in his book, diseases of the kidneys studied chiefly from a clinical and pathological point of view. To this end, preceding inflammatory diseases, is a brief résumé of the anatomy and physiology of the kidneys, which gives the reader a clear understanding of the various lesions and pathological conditions described under the various diseases of the kidneys.

Under the head of Bright's Diseases the author treats of the parenchymatous degenerations, acute and chronic, attending high temperature, infectious diseases, pregnancy, etc.; the lesions that occur in diabetes mellitus; gouty kidney; amyloid transformation, and the usual forms of acute and chronic inflammation of the kidney. Most clinicians and pathologists will disagree with him on the free use of the term. This is, however, of small importance. The diseases classed as Bright's Diseases are treated of separately and in language clear and concise, giving the pathology, the anatomical changes (gross and microscopical), the symptomatology, and the treatment consistent with modern investigation and thought. An excellent diagnostic table of Bright's Diseases is given on p. 104. A separate chapter is devoted to diabetes mellitus, and the subject is thoroughly and ably handled.

Part second is chiefly devoted to urinary analysis, chemical and microscopical.

The illustrations are all good, many of them made especially for the author. The book is a valuable addition to medical literature, and will prove an instructive companion to the practitioner and student.

The book is printed on excellent paper with large, clear type. F. B.

ON THE WASTING DISEASES OF INFANTS AND CHILDREN. By EUSTACE SMITH, M. D. Pp. xx and 380. Fifth edition. Philadelphia: P. Blakiston, Son & Company. 1888. Chicago: W. T. Keener.

The author states in the preface to this edition that the text has been revised, and many alterations and additions have been made. His reputation is so unquestionably established as an authority upon questions regarding the diseases of early life, that it goes without saying, that this is, as he intends it to be, a safe guide. It is also eminently practical, and a very desirable book to own.

